# Ingalls on Warping of Gold Plates

**Published:** 1859-04

**Authors:** D. B. Ingalls


### ARTICLE XIV.

### Ingalls *on Warping of Gold Plates.*

Clinton, Mass. *December* 15*th*, 1858.

Dr. A. M. Leslie:

*Dear Sir*—In reading your report of the discussion at a meeting of the "Western Dental Society," I find an objection to gold plate, from its liability to spring in soldering. I believe that *result* is always attributable to one, or both, of two causes, which care, and the observance of well-understood laws of expansion and contraction, can always overcome. The first cause for springing the plate is the bungling manner in which many *bed* their cases, preparatory to soldering. The second cause is a want of care in heating and cooling the work. That a set of teeth may be thrown into a lot of plaster and sand, in such a way that it would be impossible to heat the mass to a red heat without springing the plate, we all know, and we believe it is demonstrable, that they may be invested in just that quantity, so that any plate may be heated without changing it perceptibly. We have no new way to solder our teeth. The point is, the want of *care* in doing the work in the old way. Though to illustrate I will state how I preceed. I use the copper bands described in the "Dental News Letter," a few years since, by Dr. Chase, of Woodstock, Vermont. I prefer them to any other, for the ease which they can be shaped to the different cases—which is the point. In shaping the band for most cases, I place the central incisors within a quarter of an inch of the front of the band, and fetch it nearer the teeth as it goes back, so that the plaster may be the thickest at the front of the case, and gradually decrease in thickness on either side, until there is a perceptible difference in the thickness of the plaster at the molars, from what it is at the incisors; I then make a short turn, and fetch the band as near the posterior part of the plate as possible, so as to preclude the necessity of having a large body of plaster. When the plaster is thickest in front, and tapers back in symmetrical proportions, it will not crack under an even heat, and therefore removes the liability of the teeth being drawn apart. I let the plaster fill that part of the plate which is to cover the alveolar ridge, but the palatine surface of the plate I keep clean on either side. I use about equal quantities of plaster and sand to bed my teeth in. (It is at this stage of the process that I back my teeth.) When the case is ready to solder, I place it in a small portable furnace, (filled with charcoal, and well packed around the case) that I can handle when hot. It is thus kept in a position that it may have a good draught until the job is red hot; then apply the blow-pipe and flow the solder; cover up the dish to stop the draught, and let the whole cool together.

Any one can demonstrate, I think, by following these simple hints, that the springing of the plate is not a serious objection to the use of gold as a base for artificial teeth.

Respectfully, yours,

D. B. INGALLS.

[*Dent. Rev.*

